# Cross-Sectional Study on Assessment of Frequency of Intestinal Helminth Infections and Its Related Risk Factors among School Children from Adola Town, Ethiopia

**DOI:** 10.1155/2022/5908938

**Published:** 2022-04-11

**Authors:** Edaso Amana Husen, Geremew Tafesse, Sunil Tulshiram Hajare, Nitin Mahendra Chauhan, Rajesh J. Sharma, Vijay J. Upadhye

**Affiliations:** ^1^Department of Biology, College of Natural and Computational Sciences, Dilla University, Dilla 419, SNNPR, Ethiopia; ^2^Department of Biotechnology, VPASS College, Baramati, Maharashtra, India; ^3^Parul Institute of Applied Sciences, Parul University, Vadodara, 391760 Gujarat, India

## Abstract

The three main intestinal helminth infections (IHIs), ascariasis, trichuriasis, and hookworm, are common clinical disorders worldwide. These IHIs are more prevalent in tropics and subtropical countries especially in developing countries like sub-Saharan Africa responsible for morbidity, mortality, and physical as well as intellectual growth retardation in children. In Ethiopia, the burden of IHIs appears in all ages mainly due to factors like lack of education, low socio-economic status, and inadequate supply of drugs and proper awareness. The present study was carried out to access the prevalence of intestinal helminth infections and associated risk factors among school children in Adola town from Guji Zone, Oromia, Ethiopia, from August 2019 to December 2019. 404 stool samples were collected in a clean, dry, screw-capped, and wide-mouthed plastic container, fixed with 10% formal-saline solution, and transported to the Adola Hospital laboratory for further microscopic analysis by wet mount following formal ether concentration technique. Data were analyzed using SPSS version 20 by binary logistic regression model using odd ratio with 95% CI. The overall prevalence of IHIs among school children was found to be 33.91% (137/404). Rate of double infection was noted to be 2.72% (11/404). Most dominant species was *Ascaris lumbricoides* (8.9%) followed by *Hymenolepis nana* (7.7%), *Taenia saginata* (5.4%), hookworm (4.7%), *Trichuris trichiura* (2.5%), *Schistosoma mansoni* (2.2%), *Enterobius vermicularis* (1.7%), and *Strongyloides stercoralis* (0.7%), respectively. Highest prevalence was observed in male students (39.6%) compared to female students (28.8%) (*P* < 0.05). The prevalence of IHIs among school children in the age group of 6-10, 11-15, 16-20, and above 20 was 49.6%, 35.8%, 10.9%, and 3.6%, respectively (*P* < 0.05). IHI was significantly associated with some of the risk factors such as gender, educational level of students', toilet usage habit, habit of hand washing, hand washing habit before feeding and after defecation, purpose of hand washing, and awareness to intestinal helminths (*P* < 0.05). In the study area, the prevalence of IHIs is moderately high and represents a public health problem in the school children. Therefore, all stakeholders should pay attention to raise awareness about health education programs such as proper personal hygiene, environmental sanitation, improving the quality of drinking water, and treatment to reduce the consequences of intestinal helminths.

## 1. Introduction

Intestinal parasite infections are endemic diseases that are a major global health concern, particularly among children in developing countries, and can cause serious sickness in the form of acute or chronic infections [1]. Infection with parasitic worms is also a primary cause of illness and mortality in children [2]. It is also world's most important causes of physical and intellectual growth retardation [3]. Intestinal helminths (IH) are more prevalent throughout the tropics and subtropical parts of the world, especially among poor communities and in developing countries, which infected more than 3.5 billion people, of which 4.98 million years lived with disability [4]. As a result, about 300 million people suffer from severe morbidity attributed to IHIs, resulting in 10,000-135,000 deaths annually [5, 6]. Currently, it has been estimated that *Ascaris lumbricoides*, *Trichuris trichiura*, and hookworm (*Necator americanus* and *Ancylostoma duodenale*) infect 807 million, 604 million, and 576 million people worldwide, respectively [7]. In sub-Saharan Africa (SSA) especially in Ethiopia, ascariasis 26 million, hookworm 11 million, and trichuriasis 21 million people were reported to be infected, respectively [8].

The main cause of intestinal parasite infection is by soil-transmitted helminths (STH) like roundworm (*Ascaris lumbricoides*), whipworm (*Trichuris trichiura*), and hookworm (*Ancylostoma duodenale* and *Necator americanus*) [7, 9]. The majority of these diseases are caused by socio-economic factors, such as cultural practices and inadequate sanitation, and are spread by ingestion of soil-contaminated eggs, poor hygiene, undercooked diseased meat, and eating raw fruit and vegetables [10].

Infection by IHs is the major public health problem which causes chronic inflammatory disorder such as chronic anemia, growth stunting, protein-calorie malnutrition, fatigue, and poor cognitive performance; reduce long term survival; and diminished physical fitness and school attendance in school-age children [11]. Intestinal helminth infections are a major public health concern in developing nations, particularly among children in sub-Saharan Africa, Asia, and the Americas, who are afflicted with one or more helminths, such as ascariasis, trichuriasis, and hookworm [7]. This is primarily due to factors that predispose children to infections, such as poverty, poor sanitation, and malnutrition. Inadequate water supply, lack of proper sanitation, and overcrowding living conditions, combined with a lack of access to proper health care and a low level of education, make the poor particularly vulnerable to infection and disease, which frequently result in mortality and morbidity [9].

Like other developing countries, the prevalence of intestinal helminths was widely spread in Ethiopia. In Ethiopia, IHIs are highly prevalent because of poverty, low living standards, poor personal hygiene, contaminated food and water, poor environmental sanitation, poor health service providers, having an inadequate supply of drugs, and lack of adequate and proper awareness of the transmission mechanisms as well as the life cycle pattern, unsafe human waste disposal systems, inadequacy and lack of safe water supply, and low socioeconomic status [12–14].

School-age children are one of the groups that are at higher risk of IHIs [15]. The adverse effects of IHIs among children are diverse and alarming. Several studies have been conducted on the distribution and prevalence of IHIs and associated factors among school children in different parts of Ethiopia [16–19]. However, there are still several localities for which epidemiological information is not available or not yet properly documented especially Adola town, Guji Zone, Oromia, Ethiopia. There is a scarcity of enough information on the prevalence of IHI parasites from this selected area. As a result, the findings of the study will assist stakeholders and the school community in developing intervention programs, reducing illness burden by identifying risk factors, and improving children's health, environmental cleanliness, and personal hygiene. It will also aid both researchers and Adola town health officials in terms of developing periodic mass-deworming campaigns, as well as diagnosing and conducting IHI control initiatives in the area. By considering the above cause, this study was conducted to determine the prevalence of IHIs and its associated risk factors among primary school children at Adola town, Guji Zone, Oromia, Ethiopia.

## 2. Materials and Methods

### 2.1. Description of Study Area

This study was conducted on the prevalence of IHIs and its associated risk factors among school children in Adola town, Guji Zone, Oromia Regional State, Ethiopia. Adola town is located on the main road to Negele Borena at a distance of 120 km far from Guji Zone and 472 km from Addis Ababa, the capital city of Ethiopia. Based on the 2007 National Census conducted by the Central Statistical Agency of Ethiopia, this woreda has a total population of 22,938, of which 11,706 are males and 11, 232 are females. Highland (15%), middle land (55%), and low land (35%) characterize Adola town climate. The city has latitude of 6° 00′ N to 6° 05′ N and longitude of 39° 05′ E to 39° 83′ E. The average yearly temperature ranges from 15°C to 30°C, with a mean of 30°C during the study period [20]. One health facility and one hospital serve the town, with a total of 12 and 40 health staff, respectively. During data collection, 1 public health worker, 2 medical physicians, 3 nurses, 2 laboratory technicians, 1 pharmacist, and 2 health extension workers were located to carry out health center and hospital operations in order to enhance the health status of the town's residents. And also, there are 9 governmental elementary, 2 secondary, and 1 preparatory school. From nine elementary schools, namely, Bilu, Kucho, Adola, Amala Siresa, Dire Sadeka, Oda Adola, Hado Korsa, Haru Fora, and Dufa, three elementary schools, namely, Bilu, Kucho, and Adola elementary school, were selected for the proposed study.

### 2.2. Study Design

School-based cross-sectional study was conducted from August 2019 to December 2019 to determine the prevalence of IHIs and their associated risk factors among school children in three selected government elementary schools at Adola town (Adola, Bilu, and Kucho) by purposively sampling method.

### 2.3. Study Population

The study population was all students enrolled at Adola Town Primary Schools from grade 1 to 12 in 2019 G.C. In the three selected schools, a total number of enrolled students in 2019 were 4,668 students of which 2147 were males and 2521 were females.

### 2.4. Inclusion Criteria

The study was consented and enrolled students from four different age groups of Adola Elementary School, Bilu Elementary School, and Kucho Elementary School, willing to participate in accordance with the written consent, and able to give a stool sample and undergo a 30-minute face-to-face interview.

### 2.5. Exclusion Criteria

Students were removed from the study if they refused to participate in accordance with the written agreement and were not willing to give a stool sample and participate in a 30-minute face-to-face interview.

### 2.6. Sample Size Determination

The sample size of the study was determined using the single proportion population formula [21] as below.

(1) $$\begin{array}{c} n={\left(Z_{\alpha /2}\right)}^{2}*\left(P,\frac{{\left(1-P\right)}^{2}}{{\left(d\right)}^{2}}\right), \end{array}$$

where *n* is the number of sample size, *Z*_*α*/2_ is the standard normal deviate (1.96) which corresponds to 95% confidence interval (CI), *P* is the prevalence of intestinal helminths (*P* value was taken as 50%) due to lack of previous reports in the Adola town, and *d* is the precision/marginal error (*d* = 0.05) or 5%. The sample size determined for the study was 384. To minimize errors arising from the likelihood of noncompliance or dropout, the sample size was increased by 10% [16, 22]. Therefore, the final sample size used in the three elementary schools was 422.

### 2.7. Sampling Techniques

Three government elementary schools were selected among nine schools found in the Adola town using purposive sampling method due to higher number of students and near to hospital. The number of student in Adola, Bilu, and Kucho elementary schools was 2174 (*M* = 1002 and *F* = 1172), 1232 (*M* = 601 and *F* = 631), and 1262 (*M* = 544 and *F* = 718), respectively. A number of students allocated in each school were based on the total number of students in each school divided by the total number of students in the three selected primary schools. The results were decided by multiplying the calculated sample size. To select the study participants, the school children were first stratified into eight strata according to their educational level (grades 1 to 12) in the selected school. Then, from each stratum, the students were allocated for each school and grade level/each classroom by using the proportional allocation technique. Finally, the actual numbers of the study population in the study from each class were selected using a systematic sampling technique by using the class rosters as the sample frame [23–25].

### 2.8. Data Collection Instruments

Questionnaires were prepared originally in English according to the research objectives and the local situation and then translated into the Amharic and Afan Oromo languages to collect sociodemographic, environmental, and behavioral factors associated with IHIs. The researcher is requested to interview their students and filled the questionnaires. Stick, handbook, glove, and labeled stool cup were used for the data collection.

### 2.9. Collection of Stool Samples

After giving proper instruction on how to collect the stool sample, each school children were supplied the labeled and provided collection cups with unique identification numbers, clean, dry, and applicator sticks instructed to bring at least 5 g of proper fresh stool sample of his/her own separately in provided cup bottle. At the time of collection, date of sampling, number of the participant, grade, section, school name, age, and sex were recorded for each subject on a recording format, and all the specimens were then checked for their label, quantity, time, and procedure of collection. Each stool sample were emulsified in a 10% formal-saline solution and transported to Adola Hospital for laboratory examination. Finally, a portion of each of the stool samples was processed and examined by using direct wet mount under 10x-40x magnification followed by formal ether concentration techniques.

#### 2.9.1. Direct Saline Thin Layer Wet Mount Technique

After all necessary information and stool samples were collected and recorded, the fecal samples were taken to the laboratory for processing and microscopic examination using direct wet mount methods. At the laboratory, a drop of fresh physiological saline solution was placed on one end of a slide and one drop of iodine on the other side. Using an applicator stick, small portion of the fresh specimen, i.e. 0.25 mg, was picked up and mixed with a saline drop and a similar amount with the Lugols iodine. The slide was examined under the microscope for motile organisms by using low power and high power objectives to identify eggs and larvae of helminths. The number of students infected and the type of intestinal helminths were observed and recorded [26].

#### 2.9.2. Formal Ether Concentration Technique

Briefly, 3 g of fresh stool sample was suspended in 7 mL of formal saline. The suspended mixture was passed through three layers of wet cotton gauze through funnel in a centrifuge tube, and lastly, 3 mL of diethyl ether was added. The tube centrifuged rotated at 2500 rpm for 5 minutes. After centrifugation, the plug was removed with an applicator stick, and the supernatant was poured off. Two wet preparations were prepared out of the sediment after slight shaking and covered with glass cover slip. Finally, the slides were examined for the presence of parasites and type of parasites under microscope [27].

### 2.10. Study Variables

The dependent variable was detection of intestinal helminths in stool samples, and the independent variables were sociodemographic factors like sex, age, religion, educational status of students, parents' occupation, fathers' and mothers' level of education, and environmental factors (presence or absence of toilet in their homes and its usage, source and storage of water, place of defecation) and behavioral factors (hand washing before meal and after defecation, shoe wearing and fingernails habit, habit of eating improperly washed raw vegetables, eat uncooked food/vegetables, level of awareness to helminth infection).

### 2.11. Data Analysis

The data collected were coded, entered, cleaned, and analyzed using the SPSS version 20. The prevalence rate of infections was recorded in proportions. Chi-square (*χ*2) test was performed to verify the possible association between the prevalence of IHIs and categorical variables. The binary logistic regression analysis models were performed to measure the strengths of associations between the prevalence of IHIs and potential risk factors using odds ratio (crude and adjusted) with corresponding 95% confidence interval (CI). Variables which showed statistical significance during bivariate analysis at *P* value ≤ 0.25 were entered to multivariate logistic regression analysis to control potential confounders. The values were considered as statistically significant at *P* value < 0.05. The results were presented and interpreted by descriptive statistical methods such as frequency and percentage using cross tabulation and then summarizing data in the form of a graph, tables, figures, and short form of statements [28].

### 2.12. Data Quality Control

The questionnaire was prepared originally in English and then translated into the local language of Afan Oromo and Amharic. All the necessary reagents and other equipments were checked for expired date, and any incomplete questionnaires were checked by the principal investigator before examination of samples of the study subjects. Among the total positive samples, 10% were selected randomly for reexamination, and then, the selected samples were processed, examined, and cross checked by other experienced laboratory technicians who did not have information about the previous result. The result of new laboratory examination was recorded on well prepared format carefully and blindly to ensure quality control at Adola Health center.

## 3. Results

### 3.1. Sociodemographic Characteristics of Respondents

**Figure 1:**
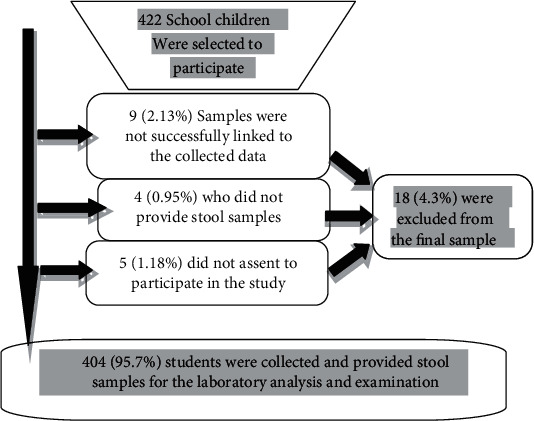
Flow chart displaying reasons for exclusion of respondents from the final sample.

In the present study, a total of 422 school students selected from Adola Town Primary Schools, 404 (95.7%) provided proper stool samples and complete information, while 18 (4.3%) students were excluded from the analysis as they fail to provide the proper stool sample or complete information (Figure 1). Among the study subjects, 192 (47.5%) were male, and 212 (52.5%) were female. The majorities (39.1%) of the study subjects were in the age group of 11-21 years old (Table 1).

### 3.2. Prevalence of IHIs with respect to Sex and Age Difference

The overall prevalence of IH infection among the study participants of Adola town was 33.91% (137 out of 404). From 404 study subjects, 39.6% males and 28.8% females were infected by either one or more intestinal helminths. There was a significant difference in the overall prevalence of IHI among males and females (*χ*2 = 5.253, df = 1, *P* = 0.022) (Table 2). Among the positive age groups, the highest prevalence was 48.2% (68/141) for those between 6 and 10 years, 31% (49/158) for children between 11 and 15 years, 19.7% (15/76) for age group of 16-20 years, and the least was 17.2% (5/29) for children above 20 years. There was statistically significant association between different age group in the prevalence of IHIs (*χ*2 = 23.895, df = 3, *P* = 0.001) (Table 2).

### 3.3. Major Intestinal Helminth Parasites Identified in Adola

In this study, eight different species of IHI organisms including *Ascaris lumbricoides*, *Hymenolepis nana*, *Taenia saginata*, hookworm, *Trichuris trichiura*, *Schistosoma mansoni*, *Enterobius vermicularis*, and *Strongyloides stercoralis* were identified by formal ether concentration technique with the prevalence rate of 36 (8.9%), 31 (7.7%), 22 (5.4%), 19 (4.7%), 10 (2.5%), 9 (2.2%), 7 (1.7%), and 3 (0.7%), respectively (Table 3). In the study area, multiple parasite infections like nematodes, cestodes, and trematodes with the prevalence of 75 (18.56%), 53 (13.12%), and 9 (2.23%) were also identified, respectively. The overall prevalence rate of double infection was 2.72%, with male and female rates of double infection of 3.65% and 1.89%, respectively (Table 3).

### 3.4. Association of IHIs with Risk Factors

The separate bivariate logistic regression at *P* ≤ 0.25 showed that schools, sex, age, educational level of students, educational level of mother, father occupation, source of drinking water, status of water container covered, treatment of water, toilet usage habit, hand washing habit, hand washing before feeding, hand washing after defecation, purpose of hand washing, and awareness about IHIs were statistically significantly (Tables 4 and 5). After adjustment of significant variables to multivariate analysis, the results showed that sex, educational level of students, toilet usage habit, hand washing habit, purpose of hand washing, hand washing before feeding, hand washing after defecation, and awareness to intestinal helminths were significantly associated with IHIs at *P* < 0.05 (Tables 4 and 5).

## 4. Discussion

Intestinal helminths are the most common infectious agents of humans in developing countries and produce a global burden of disease. Knowledge of the epidemiological, transmission, distribution, and extent of IHIs and associated risk factors of in school children are essential to design, planning, and evaluating appropriate intervention strategies [29]. Therefore, the present study was aimed at examining the prevalence of IHIs and associated risk factors in school children of Adola town, Guji Zone, Oromia, Ethiopia.

As the present study showed that the overall prevalence of IHIs recorded was 33.91%. This finding is almost comparable with the study done in Babile town, eastern Ethiopia, 27.2% [16] and Nepal [30]. On the other hand, the prevalence of IHIs observed in the current study was higher than other studies done 12.68% in MedebayZana, Tigray [31], and 13.8% in Babile town [13]. Such a relatively high prevalence of IHs is mostly due to difference in timing, seasonal and year of conducting the study, sampling of study participants, sociodemographic factors, personal hygiene practice, low levels of education, awareness about transmission and prevention of parasite, poor health services, and environmental conditions in these study areas.

However, the result of this study was much lower than the findings reported in other parts of Ethiopia, for example, 41.46% in Enderta, Tigray [32]; 54.5% in Lumame town [33]; and 65.5% in Dona Berber, Bahir Dar [34]. These variations might be due to the differences in climatic conditions, topographic, socio-economic conditions, study populations, and period in which the communities would improve their living standards, individual behavioral habits of children, and personal and environmental hygiene through time.

The prevalence rate of double parasitism was 2.72 percent, which is nearly similar to the prevalence rates reported by Ragunathan et al. [35], Ruth et al. [36], and Hailu and Ayele [37], which were 1.8 percent, 1.2 percent, and 0.9 percent, respectively. In contrast, our prevalence rate did not coincide with those of certain previous Ethiopian research which reported a greater prevalence rate [25, 31]. The most common parasitic relationship was *A. lumbricoides* + *T. trichiura* (0.99 percent), which could be owing to the similarities of their transmission pathways (feco-oral) as well as their colonization of different areas, in the digestive system [38].

The nematode helminth (18.56%) was more dominant than cestodes (13.12%) and trematodes (2.36%) in the study area. This could be due to the dry and hot weather in addition to other risk factors which facilitates the spread of nematodes faster than cestodes and trematodes, which requires high humidity and low temperatures. *Ascaris lumbricoides* (8.9%), *Hymenolepis nana* (7.7%), *Taenia saginata* (5.4%), hookworm (4.7%), *Trichuris trichiura* (2.5%), *Schistosoma mansoni* (2.2%), *Enterobius vermicularis* (1.7%), and *Strongyloides stercoralis* (0.7%) were the most common parasites identified in the present study. The predominant IH parasite species detected in the present study was *Ascaris lumbricoides* with a prevalence of 36 (8.9%). This finding is relatively lower to the study conducted in Tigray (11.5%) [32] and 10.5% in Chiro Town, west Hararghe [39]. The predominance of this parasite probably due to the easy mode of transmission of the parasite which is usually found in food, water, soil, or contaminated surface with the feces. The prevalence rate recorded in this study was also found much lower than that reported in other regions of Ethiopia 13.6% in Dona Berber [33], 37.2% in Bahir Dar [34], 48% in Bushulo village, southern Ethiopia [18], 19.1% in North Gondar [17], 27.6% in a Jimma Town [40], and higher than Tadesse [16] with the prevalence rate of 3.9% in Babile town, Eastern Ethiopia. This may be due to gregarious behavior of children while playing with poor hygienic conditions and the easy transmission way of the parasite. These observations are supported by findings of Ali [41] and Pullan and Brooker [42].

The second predominant of IHIs was *Hymenolepis nana* with the prevalence rate of 31 (7.67%) in the present study less than Babile town, Eastern Ethiopia, 10.1% [16], Medebay Zana, 11.5% [31], and Eastern Ethiopia, 13% [13]. The low prevalence of *Hymenolepis nana* might be due to climatic conditions which are crucial factor for the transmission of IHs with adequate moisture and warm temperatures [29]. Another prevalent parasites were reported in our study were *Taenia saginata* with a prevalence of 22 (5.45%), hookworm of 19 (4.7%), and *Trichuris trichiura* of 10 (2.48%). This was similar with the study done in South East, Nigeria [43], and Tigray, Ethiopia [32]. However, our results are unlike with the studies done in Homesha town, Western Ethiopia [44]. This prevalence might be due to variation in the degree of environmental contamination, local personal hygienic, and sanitary conditions.

Binary logistic regression analysis model revealed that seven determinant variables such as sex, educational level of students, toilet usage habit, presence of hand washing, hand washing after using defecation, and awareness to IHIs were significantly associated with the prevalence of the helminth parasite infection (*P* < 0.05). In this study, significantly higher prevalence of intestinal parasites was observed among male (39.6%) students than female (28.8%) (AOR = 0.566; 95% CI = 0.345-0.928; *P* = 0.024) (Table 4). This observation is supported by studies done in Babile town [16], Chiro, West Hararghe [39], Dona Berber, Bahir Dar [34]. This difference in infection rate might be due to males have more outdoor work activities in villages which carried out bare-footed, swimming behavior, working on irrigated agricultural farm lands, and expose to the more unhygienic environment [13, 29].

The age of students found one of the risk factor of IHIs. In our study, high rate of infection was recorded in the lower grade (1-4) students compared to of higher grade (5-8) (AOR = 0.039; 95% CI = 0.002-0.738; *P* = 0.031). This is consistent with previous studies done in Ethiopia [13, 16] and elsewhere [45–47]. This might be due to increased awareness about personal hygiene in higher grade (5-8) than lower grade (1-4). The observation of present study indicates that the odds of students whose toilet usage habit were 32.5% more likely to acquire IHIs than who used toilet regularly (AOR = 1.325; 95% CI = 0.109-2.966; *P* = 0.043). Similarly, those who did not have toilet usage were 34.4% (AOR = 1.344; 95% CI = 0.117-2.011; *P* = 0.052) more to be infected with the parasite as compared to toilet usage habit always.

Intestinal parasite infection was more common in children who did not wash their hands after playing or defecating and before eating, according to a study conducted in Delgi North Gondar, Ethiopia (AOR = 1.816; 95% CI = 0.992-3.323; *P* = 0.049) [17] and Kenya [48]. 80.1% of the population (AOR = 1.801; 95% CI = 0.979-3.314; *P* = 0047) in comparison to their counterparts, students who were infected with IHs did not understand the purpose of hand washing, and this was owing to a lack of proper water availability, which resulted in a high parasite infection rate [16, 49]. Furthermore, the results of the current study revealed that students who had no awareness or understanding of IHIs were 76.3% likely to be infected with IHs (AOR = 1.763; 95% CI = 1.046-2.974; *P* = 0.033). This is consistent with research published in Lumame, Ethiopia [33], and Pakistan [41]. Our study also demonstrated that the students who used uncooked vegetables and fruits were more infected, i.e., 34%, when compared to those who do not eat uncooked vegetables and fruits, i.e., 33%. However, the habit of eating raw foods was found to be statistically insignificant with infection. Similar reports were reported from North Western Tigray, Ethiopia, and from Lumane town, Northwest, Ethiopia [31, 50].

## 5. Conclusion

Unawareness of IHIs, toilet usage habits, the presence and purpose of hand washing, hand washing before feeding, and hand washing after defecation were all found to be linked with IHIs in the research population. The high frequency of IHIs is clearly linked to sociodemographic, environmental, and behavioral variables, according to the current data. The findings of this study will assist the government in focusing on infected areas and improving sanitation to reduce IHI transmission in children and their families, perhaps lowering the burden of parasite diseases in school-aged children.

### 5.1. Limitations of the Study

The proposed study has some limitations. First, the study was only limited to school children from Adola town of Ethiopia. Adding school children outside of Adola town will be handy to get a bigger picture of the prevalence of IPIs from Oromia Zone of Ethiopia. Second, the study did not identify the actual cause of splenomegaly and hepatomegaly in school children those who were positive for *S. mansoni* infection due to lack of suitable facility and financial constraints. Third, due to unavailable of state-of-the-art resources, we were unable to perform molecular techniques like PCR to identify the different intestinal helminth. Also, we were unable to refer sensitive methods specific for some intestinal parasites such as Kato-Katz method, Trichome, and modifed Ziehl–Neelsen staining methods to identify intestinal parasites. Lastly, the study did not include anthropometric measurements due to resources and budget scarcity. However, to our knowledge, this study is the first approach to determine the prevalence and associated risk factors for IPIs among school children from Adola town of Ethiopia.

## Figures and Tables

**Table 1:** Sociodemographic characteristics with respect to their prevalence of IHs among school children from Adola, Ethiopia (*n* = 404).

Variables	Categories	Total number of examined, *N* (%)	Intestinal helminths				
			Positive, *N* (%)	Negative, *N* (%)	*χ*2	Df	*P* value
School	Adola	188 (46.5)	50 (26.6)	138 (73.4)	8.404	2	0.015
	Bilu	106 (26.2)	43 (40.6)	63 (59.4)			
	Kucho	110 (27.2)	44 (40)	66 (60)			
Sex	Male	192 (47.5)	76 (39.6)	116 (60.4)	5.253	1	0.022
	Female	212 (52.5)	61 (28.8)	151 (71.2)			
Age (Years)	6-10	141 (34.9)	68 (48.2)	73 (51.8)	23.895	3	0.001
	11-16	158 (39.1)	49 (31)	109 (69)			
	16-20	76 (18.8)	15 (19.7)	61 (80.3)			
	>20	29 (7.2)	5 (17.2)	24 (82.8)			
Ethnicity	Oromo	359 (88.9)	123 (34.3)	236 (65.7)	1.480	3	0.687
	Amhara	9 (2.2)	3 (33.3)	6 (66.6)			
	SNNP	21 (5.2)	8 (38)	13 (62)			
	Other	15 (3.7)	3 (20)	12 (80)			
Religion	Muslim	101 (25.0)	30 (29.7)	71 (70.3)	3.589	3	0.309
	Orthodox	41 (10.1)	10 (24.4)	31 (75.6)			
	Protestant	243 (60.1)	90 (37)	153 (63)			
	Catholic	19 (4.7)	7 (36.8)	12 (63.2)			
Educational level of students	Grades 1-4	175 (43.3)	86 (49)	89 (51)	39.396	7	0.001
	Grades 5-8	229 (56.7)	51 (22.3)	178 (77.7)			
Educational level of father	Illiterate	182 (45.0)	72 (39.6)	110 (60.4)	8.928	4	0.063
	Elementary school	141 (34.9)	48 (34)	93 (66)			
	Secondary and preparatory	44 (10.9)	10 (22.7)	34 (77.3)			
	College	29 (7.2)	5 (17.2)	24 (82.8)			
	University and above	8 (2.0)	2 (25)	6 (75)			
Educational level of mother	Illiterate	216 (53.5)	84 (61.3)	132 (49.4)	6.482	4	0.166
	Elementary school	101 (25.0)	31 (30.7)	70 (69.3)			
	Secondary and preparatory	52 (12.9)	12 (23)	40 (77)			
	College	29 (7.2)	9 (31)	20 (69)			
	University and above	6 (1.5)	1 (17)	5 (83)			
Father occupation	Merchant	62 (15.3)	20 (32.3)	42 (67.7)	9.844	4	0.043
	Daily laborer	85 (21.0)	25 (29.4)	60 (70.6)			
	Government employee	32 (7.9)	7 (22)	25 (78)			
	Private employee	53 (13.1)	13 (24.5)	40 (75.5)			
	Farmer	172 (42.6)	72 (42)	100 (58)			
Mother occupation	Merchant	61 (15.1)	20 (32.8)	41 (67.2)	2.697	4	0.610
	Daily laborer	83 (20.5)	24 (28.9)	59 (71.1)			
	Government employee	37 (9.2)	12 (32.4)	25 (67.6)			
	Private employee	73 (18.1)	23 (31.5)	50 (68.5)			
	Housewife	150 (37.1)	58 (38.7)	92 (61.3)			

**Table 2:** Prevalence of IHIs by age and sex among school children from Adola, Ethiopia.

Age group of students		Sex of students				Both sex		*χ*2	Df	*P* value
		Male		Female						
		Examined no	Positive, no. (%)	Examined no.	Positive, no. (%)	Examined no.	Positive, no. (%)			
	6-10	66	39 (59.1)	75	29 (38.7)	141	68 (48.2)	3.521	3	0.318
	11-15	68	23 (33.8)	90	26 (28.9)	158	49 (31)			
	16-20	45	11 (24.4)	31	4 (13)	76	15 (19.7)			
	Above 20	13	3 (23)	16	2 (12.5)	29	5 (17.2)			
	Total	192	76 (39.6)	212	61 (28.8)	404	137 (33.91)			

**Table 3:** Distribution of IH species among school children from Adola, Ethiopia.

Types of helminth infection	Parasite species	Prevalence of IH students by sex		
		Male (*n* = 192)	Female (*n* = 212)	Total (*n* = 404)
		No. (%)	No. (%)	No. (%)
Single infection				
Nematodes (roundworms)	AL	22 (11.46)	14 (6.60)	36 (8.91)
	TT	7 (3.65)	3 (1.42)	10 (2.48)
	SS	1 (0.52)	2 (0.94)	3 (0.74)
	HW	11 (5.73)	8 (3.77)	19 (4.70)
	EV	3 (1.56)	4 (1.89)	7 (1.73)
	Total	44 (22.92)	31 (14.62)	75 (18.56)
Trematodes (flukes)	SM	4 (2.08)	5 (2.36)	9 (2.23)
	Total	4 (2.08)	5 (2.36)	9 (2.23)
Cestodes (tapeworms)	TS	15 (7.81)	7 (3.30)	22 (5.45)
	HN	13 (6.77)	18 (8.49)	31 (7.67)
	Total	28 (14.88)	25 (11.79)	53 (13.12)
Double infection				
AL + TT		3 (1.56)	1 (0.47)	4 (0.99)
AL + HW		1 (0.52)	1 (0.47)	2 (0.50)
AL + TS		2 (1.04)	1 (0.47)	3 (0.74)
HW + TT		0 (0.00)	1 (0.47)	1 (0.25)
HW + TS		1 (0.52)	0 (0.00)	1 (0.25)
Total		7 (3.65)	4 (1.89)	11 (2.72)

^∗^AL: Ascaris lumbricoides; EV: Enterobius vermicularis; HN: Hymenolepis nana; HW: hookworm; SM: Schistosoma mansoni; SS: Strongyloides stercoralis; TS: Taenia saginata; TT: Trichuris trichiura.

**Table 4:** Bivariate and multivariate analysis association of IHIs with sociodemographic factors among school children from Adola, Ethiopia.

Variables categories		Total examined, no. (%)	Intestinal helminths		COR (95% CI)	AOR (95% CI)	*P* value
			Positive, *N* (%)	Negative, *N* (%)			
Schools	Adola	188 (46.5)	50 (26.6)	138 (73.4)	1	1	
	Bilu	106 (26.2)	43 (40.6)	63 (59.4)	1.840 (1.116-3.034)	1.105 (0.588-2.076)	0.757
	Kucho	110 (27.2)	44 (40)	66 (60)	0.977 (0.567-1.683)	0.994 (0.529-1.87)	0.986
Sex	Male	192 (47.5)	76 (39.6)	116 (60.4)	1	1	
	Female	212 (52.5)	61 (28.8)	151 (71.2)	0.617 (0.407-0.934)	0.566 (0.345-0.928)	0.024^∗^
Age (years)	6-10	141 (34.9)	68 (48.2)	73 (51.8)	1	1	
	11-16	158 (39.1)	49 (31)	109 (69)	0.224 (0.081-0.619)	9.306 (0.465-186)	0.144
	16-20	76 (18.8)	15 (19.7)	61 (80.3)	0.463 (0.167-1.286)	2.236 (0.412-12.187)	0.352
	>20	29 (7.2)	5 (17.2)	24 (82.8)	0.847 (0.277-2.589)	1.986 (0.459-8.596)	0.359
Educational level of students	Grades 1-4	175 (43.3)	86 (49)	89 (51)	1	1	
	Grades 5-8	229 (56.7)	51 (22.3)	178 (77.7)	0.156 (0.058-0.421)	0.039 (0.002-0.738)	0.031^∗^
	Illiterate	182 (45.0)	72 (39.6)	110 (60.4)	1	1	0.063
	Elementary	141 (34.9)	48 (34)	93 (66)	1.497 (0.829-2.706)	1.754 (0.799-3.853)	
Education level of father	Secondary and preparatory	44 (10.9)	10 (22.7)	34 (77.3)	1.318 (0.501-3.463)	1.411 (0.427-4.659)	
	College	29 (7.2)	5 (17.2)	24 (82.8)	0.689 (0.613-2.914)	0725 (0.996-4.047)	
	University and above	8 (2.0)	2 (25)	6 (75)	0.645 (0.125-3.122)	0.734 (0.216-2.493)	
Educational level of mother	Illiterate	216 (53.5)	84 (61.3)	132 (49.4)	1	1	
	Elementary	101 (25.0)	31 (30.7)	70 (69.3)	0.314 (0.036-2.737)	0.828 (0.073-9.418)	0.879
	Secondary higher	52 (12.9)	12 (23)	40 (77)	0.452 (0.051-4.028)	0.810 (0.071-9.230)	0.865
	Diploma	29 (7.2)	9 (31)	20 (69)	0.667 (0.071-6.274)	1.043 (0.088-12.362)	0.973
	Degree and above	6 (1.5)	1 (17)	5 (83)	0.444 (0.045-4.374)	0.852 (0.066-10.969)	0.902
Father occupation	Merchant	62 (15.3)	20 (32.3)	42 (67.7)	1	1	
	Daily laborer	85 (21.0)	25 (29.4)	60 (70.6)	1.512 (0.819-2.790)	1.314 (0.606-2.851)	0.489
	Government employee	32 (7.9)	7 (22)	25 (78)	1.728 (0.991-3.014)	1.368 (0.701-2.672)	0.358
	Private employee	53 (13.1)	13 (24.5)	40 (75.5)	2.571 (1.055-6.269)	0.532 (0.076-3.709)	0.524
	Farmer	172 (42.6)	72 (42)	100 (58)	2.215 (1.105-4.440)	1.605 (0.679-3.792)	0.281

^∗^Statistically significant.

**Table 5:** Bivariate and multivariate analysis association between behavioral and environmental factors with prevalence of IHIs among school children from Adola, Ethiopia.

Categories		Total examined, *n* = 404, *N* (%)	Intestinal helminths		COR (95% CI)	AOR (95% CI)	*P* value
			Positive, *n* = 137, *N* (%)	Negative, *n* = 267, *N* (%)			
Source of drinking water							
	River	68 (16.83)	19 (28)	49 (72)	1	1	
	Stream	147 (36.39)	59 (40)	88 (60)	1.172 (0.359-3.824)	2.627 (0.555-12.447)	0.224
	Tap	173 (42.82)	54 (31.2)	119 (68.8)	0.678 (0.224-2.052)	1.594 (0.348-7.306)	0.548
	Earth dam	16 (3.96)	5 (31)	11 (69)	1.002 (0.332-3.024)	2.593 (0.586-11.466)	0.209
Status of water container covered							
	Covered	287 (71.04)	92 (32)	195 (68)	1	1	
	Uncovered	117 (28.96)	45 (38.5)	72 (61.5)	1.325 (0.847-2.072)	0.888 (0.470-1.678)	0.714
Treatment of water							
	Yes	296 (73.27)	92 (31)	204 (69)	1	1	
	No	108 (26.73)	45 (41.7)	63 (58.3)	1.584 (1.005-2.496)	1.689 (0.978-2.915)	0.060
Toilet usage habit							
	Always	192 (47.52)	57 (30)	135 (70)	1	1	
	Sometimes	130 (32.18)	48 (37)	82 (63)	0.339 (0.135-0.850)	1.325 (0.109-2.966)	0.043^∗^
	Never	82 (20.30)	32 (39)	50 (61)	0.310 (0.123-0.779)	1.344 (0.117-2.011)	0.052
Hand washing habit							
	Yes	283 (70.05)	82 (29)	201 (71)	1	1	
	No	121 (29.95)	55 (45.5)	66 (54.5)	2.043 (1.315-3.173)	1.816 (0.992-3.323)	0.049^∗^
Hand washing before feeding							
	Yes	200 (49.40)	54 (27)	146 (73)	1	1	
	No	204 (50.50)	28 (13.72)	176 (86.27)	1.967 (1.302-2.972)	2.324 (1.401-3.858)	0.001^∗^
Hand washing after defecation							
	Yes	211 (52.33)	61 (28.9)	150 (71.1)	1	1	
	No	193 (47.77)	21 (10.88)	172 (89.11)	2.657 (1.684-4.191)	3.330 (1.937-5.728)	0.001^∗^
Purpose of hand washing							
	Know	255 (63.12)	80 (31.4)	175 (68.6)	1	1	
	Unknown	149 (36.88)	57 (38.3)	92 (61.7)	1.355 (0.888-2.069)	1.801 (0.979-3.314)	0.047^∗^
	Habit of eating uncooked food or vegetables						
	Yes	225 (55.69)	78 (34.6)	147 (65.3)	1	1	0.719
	No	179 (44.31)	59 (32.9)	120 (67)	0.950 (0.643-1.405)	1.079 (0.712-1.635)	
Awareness about IHIs							
	Yes	205 (50.74)	58 (28.3)	147 (71.7)	1	1	
	No	199 (49.26)	79 (40)	120 (60)	1.669 (1.101-2.529)	1.763 (1.046-2.974)	0.033^∗^

^∗^Statistically significant.

## Data Availability

Not applicable. However, raw data can be obtained from the corresponding author upon kind request.
